# Exploring Public Knowledge, Attitudes, and Barriers to Using Genetic Services in Damanhur City and Beni-Suef City, Egypt: A Cross-Sectional Study

**DOI:** 10.7759/cureus.57171

**Published:** 2024-03-29

**Authors:** Sameer H Hafez, Noha A Mohammed, Ahmad A. Alshehri, Mohammed Khalid Hussein, Hanan Saad Abdullah Alwadei, Elsadig Eltaher Hamed Abdulrahman, Samah Ramadan Elrefaey, Amal Elhaj Alawad, Nahla Elradhi Abdulrahman, Mohamed Saied Harfoush

**Affiliations:** 1 Community Health Nursing, Beni-Suef University, Beni-Suef, EGY; 2 Clinical Laboratory Sciences, Faculty of Applied Medical Sciences, Najran University, Najran, SAU; 3 Medical Surgical Nursing, Faculty of Nursing, Jazan University, Jazan, SAU; 4 Community and Mental Health Nursing, Faculty of Nursing, Najran University, Najran, SAU; 5 Medical and Surgical Nursing, Faculty of Nursing, Najran University, Najran, SAU; 6 Psychiatric and Mental Health Nursing, Faculty of Nursing, Benha University, Benha, EGY; 7 Microbiology, College of Medicine, Najran University, Najran, SAU; 8 Community Health Nursing, Faculty of Nursing, Damanhour University, Damanhour, EGY; 9 Nursing, Buraydah College for Applied Medical Sciences, Buraydah, SAU

**Keywords:** perceived barriers, egyptian population, attitude, awareness, genetic services

## Abstract

Background: Advancements in genetic disorder management mark a transformative era in healthcare. This study aimed to assess knowledge, attitudes, and barriers to using genetic services among the Egyptian population.

Methods: A cross-sectional study was used to achieve the aim of the study. A convenient sample was used to involve 385 residents of Damanhur City and Beni-Suef City to represent Upper and Lower Egypt. A validated questionnaire covering socio-demographic details, genetic knowledge, attitudes, and perceived barriers to using genetic services was used.

Results: Regarding genetic knowledge, 70.9% of the participants reported an unsatisfactory level of knowledge about genetics. Furthermore, 67.6% expressed a negative attitude toward genetic services. Concerns about whether the test result is positive were the most common obstacle, cited by 64.94% of participants, followed by cost, which 60.78% of people found to be a major barrier. Significant associations emerge between socio-demographic factors and awareness levels.

Conclusion: The findings illuminate significant gaps in knowledge and attitude levels where less than a third of the participants had a satisfactory level of knowledge and about one-third had a positive attitude regarding genetic testing. Barriers such as concerns about treatment strategies, financial constraints, and conflict with personal beliefs emerge as critical obstacles. The identified associations between socio-demographic factors and awareness levels underscore the need for targeted interventions tailored to specific demographic groups.

Recommendations: This study recommends developing and implementing culturally sensitive awareness campaigns about genetics tailored to the specific demographic characteristics of the Egyptian population.

## Introduction

Around the world, people struggle with the lifelong effects of genetic diseases. Thanks in part to the efforts of communities, scientists, patients, and extensive research, approved gene therapies are now available to treat certain genetic diseases. The goal of research is to eventually develop even more treatments for a wider variety of genetic diseases [1].

A genetic condition results from abnormalities in one or more genes or chromosomes and may include changes in a single gene, the addition/deletion of an entire chromosome, or changes in multiple chromosomes [2]. Environmental factors and complex gene interactions contribute to the development of multifactorial genetic diseases. The Arab community faces a higher prevalence of genetic diseases. In Egypt, genetic diseases continue to be a significant contributor to morbidity, mortality, and disability, reflecting their widespread occurrence [3].

Advances in genomic science have shifted the focus of healthcare from the mere diagnosis and treatment of genetic diseases to include genetic risk assessment, counseling, and preventive measures. Expanding capabilities in the diagnosis and treatment of genetic disorders have increased the demand for genetic services. These services are accessible through various avenues, such as newborn screening programs, where all infants are tested for a spectrum of genetic disorders immediately after birth. In addition, clinical diagnostic options are available that incorporate the expertise of geneticists, neurologists, oncologists, and other specialists [4].

Genetic counseling, a specialized field, focuses on identifying individuals at risk of genetic disorders. Following genetic testing, patients and family members are recommended for additional screening and risk reduction methods. Despite the potential benefits, there is a deficiency in the provision of genetic counseling [5]. Studies have highlighted disparities in awareness, with racial/ethnic minorities and rural populations being less informed about testing options, underscoring the underserved nature of rural communities [6]. As genetic counseling and testing become integral components of clinical care, it is critical to tailor genetic services to the cultural values of communities, as these values significantly influence the perception of genetic services [7,8].

Egypt faces significant economic and healthcare challenges due to overpopulation. Genomic and genetic testing have emerged as substantial challenges in this context. Egypt underscored the importance of advancing medical genetics and genomics nationwide to address prevalent genetic diseases effectively. To achieve this, there is a pressing need for comprehensive knowledge of the Egyptian genome and associated diseases and for understanding the perspectives of the population regarding the use of genetic services [9].

Objectives and research questions

This study aims to assess the knowledge, attitudes, and barriers to using genetic services among the Egyptian population. The research questions are the following: What are the participants' levels of knowledge regarding general genetics? What are the barriers experienced by the participant regarding the use of genetic services? What are the participants' levels of attitude regarding the use of genetic services?

## Materials and methods

Research design and study setting

The research employed a cross-sectional study design to comprehensively investigate the Egyptian public's knowledge, attitudes, and barriers concerning the utilization of genetic services. The study was conducted in Damanhur City, Beheira Governorate, and Beni-Suef City, Beni-Suef Governorate, to represent Upper and Lower Egypt. The data was collected by interviewing the subjects in primary healthcare centers and general places.

Sample size and sampling technique

The convenience sample technique was used to involve a sample size of 385 participants, determined through a systematic calculation using the formula: sample size = (1.962) x (50) x (1-0.5) / 0.052. This formula, commonly known as the sample size estimation formula, takes into account factors such as the desired confidence level (1.96 for a 95% confidence interval), the margin of error (0.052), and the estimated proportion of the population with a particular characteristic (in this case, 50%).

Inclusion and exclusion criteria

The inclusion criteria for this study encompassed individuals aged 18 years and older who willingly agreed to participate in the research. The target population included residents of Damanhur City, Beheira Governorate, and Beni-Suef City, Beni-Suef Governorate, Egypt. Participants were selected without discrimination based on gender, educational background, or socioeconomic status. The exclusion criteria comprised individuals below the age of 18, those who declined to participate, and those residing outside the specified geographical area. The rationale behind these criteria was to ensure a focused and representative sample for the examination of the Egyptian public's knowledge, attitudes, and barriers regarding genetic services in the context of genetic disorder management within the designated region.

Data collection tools

The data collection tools for this study consisted of a comprehensive questionnaire designed by the researcher after a thorough review of relevant literature. The structured questionnaire comprised four distinct parts. The first part focused on gathering socio-demographic information from the participants, including details such as age, gender, educational level, and residence. The second part of the questionnaire assessed the participants' general knowledge of genetics using 15 structured true or false questions. These questions covered fundamental genetic concepts, the inheritance of diseases, gene-environment interactions, and genetic variation. The questions were adapted from existing literature [10]. A scoring system was employed, with each correct answer receiving a score of "1" and incorrect answers marked as "0." The total score for multiple-choice questions was 15, and the results were converted into percentages, categorized as unsatisfactory (<60%) or satisfactory (≥60%).

The third part of the questionnaire delved into participants' attitudes toward the utilization of genetic services. Comprising eight items on a scale of 1 to 3, responses ranged from agreement to disagreement. The items were rephrased from previous studies [11-13] with some modifications. The total attitude score ranged from 1 to 24, and respondents scoring 60% or above were considered to have a positive attitude toward genetic testing. The fourth part focused on a barriers questionnaire designed by the researchers based on the existing literature to assess challenges and barriers reported by the participants. These barriers included cost, lack of clear treatment strategies, worries about positive test results, absence of specialized centers, confusion over testing options, and conflicts with beliefs in fate and destiny. Participants' responses were scored as "1" if the barrier was present and "0" if not. To ensure the reliability and validity of the questionnaire, it underwent validation by five professors in the community health nursing and medical-surgical nursing departments.

Validity and reliability of the study

The consistency, as measured by Cronbach's alpha coefficient test, was 0.78 for the knowledge section and 0.84 for the attitude section in the current study, attesting to the reliability of the data collection instruments. A panel of five professors specializing in community health nursing and medical-surgical nursing departments undertook the validation process, bringing diverse expertise to the evaluation.

Field of the work

The data collection phase spanned from October 2022 to May 2023, encompassing a robust timeframe to capture a comprehensive snapshot of the Egyptian public's awareness, attitudes, and barriers regarding genetic services. The researcher randomly selected two maternal and child health centers from each city to involve the study participants. The researchers provided information about the study objective, procedures, and potential benefits and then obtained informed consent from willing participants before proceeding with data collection. The interview was done with the participants in a private and confidential setting at the health centers, and we addressed any queries they may have.

Ethical considerations

Approval was obtained from the Research Ethics Committee of the Faculty of Nursing at Damanhur University under code number 18/8/2022/59-d. The study was conducted with careful attention to the ethical standards of research and the rights of participants. At first, verbal consent from the participants was obtained. The participants were informed about the purpose of the study, and they had the right to refuse to participate. The anonymity of the participants was maintained at all times.

Statistical analysis

The comprehensive analysis of research participants involved a meticulous exploration of various facets, including demographics, specific responses to tailored questions, as well as their knowledge and attitudes toward genetics. Descriptive statistics formed the bedrock of this exploration, offering a detailed portrayal of the study cohort. The SPSS Statistics version 24.0 (IBM Corp. Released 2016. IBM SPSS Statistics for Windows, Version 24.0. Armonk, NY: IBM Corp.) served as the analytical powerhouse, facilitating the extraction of meaningful insights from the amassed data. The application of the Chi-square test, a robust statistical tool, played a pivotal role in discerning significant differences between the variables under scrutiny. A Spearman correlation test was used to measure the correlation between the knowledge score and attitude score of the studied sample. The benchmark for statistical significance was set at a p-value less than 0.05, ensuring that the findings were robust and could be considered as meaningful indicators of patterns, associations, or differentials within the dataset.

## Results

The frequency distribution in Table 1 provides a snapshot of the socio-demographic characteristics of the sample studied. The age distribution shows a relatively balanced representation, with 150 (38.96%) of the participants under 30 years old, 120 (31.17%) between 30 and 50 years old, and 115 (29.87%) over 50 years old. In terms of gender, 210 (54.5%) of the participants are male. Educational levels are diverse, with 98 (25.45%) with primary education, 110 (28.57%) with secondary education, 100 (25.97%) with university education, and 77 (20%) with higher education (master's and Ph.D.). In terms of place of residence, a significant majority of 313 (81.30%) live in rural areas, while 72 (18.70%) live in urban areas. This detailed breakdown of socio-demographic characteristics lays the groundwork for a nuanced understanding of the study population and provides essential context for interpreting subsequent findings related to awareness, attitudes, and barriers to genetic services among the Egyptian public.

**Table 1:** Frequency distribution of studied sample regarding socio-demographic traits

Items	N	%
Age		
<30	150	38.96
30-50	120	31.17
>50	115	29.87
Gender		
Male	210	54.55
Female	175	45.45
Educational levels		
Primary	98	25.45
Secondary	110	28.57
University education	100	25.97
Higher education	77	20
Residence		
Rural	313	81.30
Urban	72	18.70

Table 2 shows the frequency distribution of the studied sample regarding their history of genetic problems and involvement in genetic testing. The results show that 82 (21.3%) of the participants report a family history of genetic diseases, while 201 (52.8%) don't know. The results show that of the 385 participants, 92 (23.9%) reported undergoing genetic testing, while 293 (76.1%) did not. Among those who underwent genetic testing (92 participants), premarital screening was the most common type of testing, reported by 73 (79.4%) of participants who underwent genetic testing, while only 19 (20.6%) reported undergoing genetic screening before pregnancy. Among those who underwent genetic testing, only 18 (19.6%) received genetic counseling either before or after testing.

**Table 2:** Frequency distribution of family history and genetic testing among the studied sample

Items	N	%
Family history of genetic diseases		
Yes	82	21.3
Don’t know	203	52.8
No	100	25.9
Performing genetic testing (385)		
Yes	92	23.9
No	293	76.1
Types of tests (92)		
Premarital screening	73	79.4
Genetic screening before pregnancy	19	20.6
Do you get genetic counseling before or after the test from the primary healthcare centers? (92		
Yes	18	19.6
No	74	80.4

The results presented in Table 3 highlight the frequency distribution of barriers to genetic services encountered by the sample. Concerns about positive test results emerged as the most common barrier, with 250 (64.94%) of participants expressing concerns in this regard. Cost was a close second, with 234 (60.78%) of participants citing financial constraints. Another notable finding was the perception of inadequate treatment strategies for genetic diseases, with 293 (76.10%) of participants expressing concern in this area. Difficulty accessing specialized centers was reported by 187 (48.57%) of participants, indicating geographic or logistical challenges in accessing genetic services.

**Table 3:** Frequency distribution of the studied sample regarding barriers to using the genetic services

Items	N	%
Concerns whether the results of the test are positive	250	64.94
Cost	234	60.78
No clear treatment strategies for genetic diseases	293	76.10
Difficulty in accessing specialized centers	187	48.57
Confusion over different testing options	98	25.45
It conflicts with the belief in fate and destiny	156	40.52

The results presented in Table 4 illustrate the frequency distribution of the surveyed participants regarding their level of knowledge of genetics. A significant proportion of participants (273, 70.9%) reported an unsatisfactory level of knowledge in this area. Conversely, 112 (29.1%) of the participants indicated a satisfactory level of knowledge regarding genetics.

**Table 4:** Frequency distribution of studied participants regarding their levels of knowledge regarding genetics

Items	Knowledge	
	N	%
Satisfactory	112	29.1
Unsatisfactory	273	70.9

Table 5 shows the frequency distribution of the studied participants regarding their level of attitude. Of the participants, 125 (32.4%) reported a positive attitude, while the majority (260, 67.6%) reported a negative attitude.

**Table 5:** Frequency distribution of studied participants regarding their levels of awareness and their levels of attitude

Items	Attitude	
	N	%
Positive	125	32.4
Negative	260	67.6

Table 6 presents the association between the socio-demographic characteristics of the studied participants and their levels of awareness. The results reveal significant associations between age, gender, educational stage, and residence with awareness levels. Specifically, participants aged 30-50 have the highest percentage related to satisfactory knowledge (X² = 5.9, p = 0.005). Gender exhibited a highly significant association (X² = 45.9, p < 0.00001), highlighting differences in awareness levels between males and females; women are more knowledgeable than men. The educational stage also demonstrated a significant association (X² = 35.4, p < 0.00001), emphasizing that participants with Ph.D. or master's degrees are more knowledgeable. Interestingly, residence (urban or rural) showed no significant association with awareness levels. These findings underscore the need for targeted awareness campaigns tailored to specific demographic groups, considering the identified associations. The table provides valuable insights into the factors influencing awareness levels, guiding efforts to enhance genetic services awareness in the Egyptian population.

**Table 6:** Association between the socio-demographic characteristics of the studied participants and their levels of awareness

Items	N	Awareness		X^2^	P
		Satisfactory (112)	Unsatisfactory (273)		
Age				5.9	0.005
18-30	150	46 (30.6%)	104 (69.4%)		
30-50	120	42 (35%)	78 (65%)		
>50	115	24 (20.8%)	91 (79.2%)		
Gender				45.9	0.00001
Male	210	31 (14.7%)	179 (85.3%)		
Female	175	81 (46.2%)	94 (53.8%)		
Educational stage				35.4	0.00001
Primary	98	15 (15.3%)	83 (84.7%)		
Secondary	110	20 (18.2)	90 (81.8%)		
University	100	39 (39.0%)	61 (61.0%)		
Ph.D./master's degree	77	38 (49.4%)	39 (50.6%)		
Residence				0.3	0.5
Rural	313	89 (28.4%)	224 (71.6%)		
Urban	72	23 (31.9%)	49 (68.1%)		

**Figure 1:**
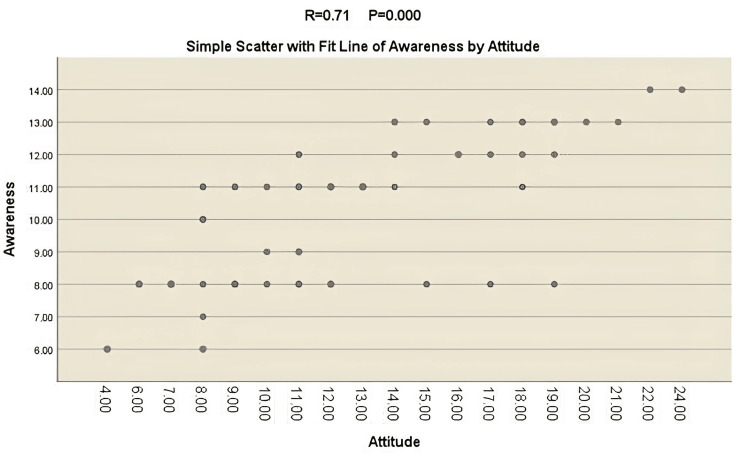
The correlation between the participants' awareness and their overall attitude

Figure 1 illustrates the correlation between participants' awareness and their overall attitudes toward genetic services. The results reveal a positive and significant correlation between participants' awareness and attitudes. This indicates that as awareness levels increase, there is a corresponding positive shift in attitudes toward genetic services. The findings emphasize the interconnectedness of awareness and attitudes, suggesting that interventions aimed at improving awareness may contribute to fostering more favorable attitudes among the Egyptian public. This positive correlation underscores the importance of comprehensive awareness campaigns to shape positive attitudes and promote the utilization of genetic services in the context of genetic disorder management in Egypt.

## Discussion

The current study aimed to assess knowledge, attitudes, and barriers to using genetic services among the Egyptian population. The study revealed that only about one-quarter underwent genetic testing. The results revealed the perceived barriers to using genetic services, such as concerns about test results, financial constraints, difficulty accessing specialized centers, and conflicts with beliefs in fate and destiny. The study's findings align with those of Raspa et al. [4], which highlighted challenges such as a lack of specialized centers, waiting times, cost issues, and services delivered by non-genetics providers in the utilization of genetic services. Similarly, Niyibizi et al. [14] identified common barriers such as concerns about positive test results, unclear post-test care pathways, cost issues, and a lack of specialized centers. In contrast, Swink et al. [5] reported that a majority of their participants appropriately used genetic counseling and testing without hesitation, with those not undergoing counseling not being physician-referred. The variation in results may be attributed to the predominantly rural residence of participants in the current study and the lack of health insurance coverage for genetic services' costs.

In the current study, approximately one-third of the participants demonstrated a satisfactory level of knowledge. These findings align with Rahma et al. [15], who reported that 29.5% of their participants had adequate awareness, while 70.5% had inadequate awareness. Notably, the awareness of the studied participants in the current research was higher than that reported by Vermeulen et al. [16] in the United Arab Emirates, where only 7% of participants exhibited a good knowledge level in genetics and genomics. Conversely, the results were lower than those reported by Alotaibi et al. [10] in Saudi Arabia, where two-thirds of the participants had sufficient knowledge of genetics. It is important to consider that a significant proportion of the Saudi participants were university students, likely exposed to genetics fundamentals through academic courses, unlike more than half of the participants in the current study, who had primary and secondary education backgrounds.

Concerning the attitude of the studied sample, approximately one-third of the studied sample had a positive attitude toward the utilization of genetic services. This finding is consistent with [17] who reported that less than half of the respondents expressed interest in genetic testing to prevent specific diseases such as cancer, cardiovascular disease, diabetes, or dementia. However, there is a discrepancy with the study by Jaya et al. [11] where the majority of participants demonstrated a positive attitude toward genetic services. Additionally, Vermeulen et al. [16] found that 76.9% of participants were willing to undergo a genetic test if their relatives had a genetic disease. The variations among these studies may be attributed to factors such as lower levels of education and cultural differences among the participants in the current study.

The current study revealed that the knowledge of the participants was significantly influenced by their age, gender, and educational level. Notably, females exhibited a higher level of knowledge compared to males, potentially attributable to the typical concern women have for their health and that of their children. Furthermore, individuals with a higher level of education demonstrated a more satisfactory level of knowledge. These findings align with the results reported by Almomani et al. and Hashemi-Soteh et al. [17,18]. Moreover, the current study found that participants with a higher level of awareness also showed a more positive attitude toward the utilization of genetic services. This correlation between awareness and attitude is consistent with the results reported by Alotaibi et al. [10], emphasizing a positive and significant association between participants' awareness and their attitudes.

Implications

The implications of the current study extend to both healthcare practitioners and policymakers, shedding light on critical aspects of the utilization of genetic services among the Egyptian public. Firstly, the findings underscore the urgent need for targeted interventions aimed at enhancing awareness and understanding of genetic services, especially among demographic groups characterized by limited awareness, such as those under 30, predominantly male, and residing in rural areas. Secondly, the study highlights the significance of addressing barriers that hinder the optimal utilization of genetic services.

Limitations

Despite the valuable insights provided by this study, it is essential to acknowledge its limitations. The study captures a snapshot of awareness, attitudes, and barriers at a specific point in time, preventing the identification of trends or changes over an extended period. Secondly, the reliance on self-reported data introduces the potential for social desirability bias, where participants may provide responses they perceive as socially acceptable. Furthermore, the study's reliance on a single data collection method, a structured questionnaire, might overlook nuanced perspectives that could be captured through qualitative methods. While the sample size was estimated according to a scientific formula, it was relatively small, and the convenience technique makes it possible to generalize the results to the Egyptian population. Despite these limitations, the study offers valuable insights into the current landscape of genetic service awareness and utilization in Egypt, providing a foundation for future research and targeted interventions.

Recommendations

This study recommends developing and implementing culturally sensitive awareness campaigns about genetics tailored to the specific demographic characteristics of the Egyptian population and directing more efforts toward the expansion and equitable distribution of specialized centers offering genetic services, particularly in rural areas.

## Conclusions

The findings illuminate significant gaps in knowledge and attitude level, where less than one-third had a satisfactory level of knowledge and about one-third had a positive attitude regarding genetic testing. Barriers such as concerns about treatment strategies, financial constraints, and conflicts with personal beliefs emerge as critical obstacles. The identified associations between socio-demographic factors and awareness levels underscore the need for targeted interventions tailored to specific demographic groups.
